# Food price elasticity estimates in Australia

**DOI:** 10.1038/s43016-025-01184-1

**Published:** 2025-07-11

**Authors:** Tazman Davies, Akshar Saxena, Jason H. Y. Wu, Matti Marklund

**Affiliations:** 1 The George Institute for Global Health, Faculty of Medicine, University of New South Wales, Sydney, NSW Australia; 2 Division of Economics, Nanyang Technological University, Singapore, Singapore; 3 Department of Epidemiology, John Hopkins Bloomberg School of Public Health, Baltimore, MD USA; 4 Department of Public Health and Caring Sciences, Clinical Nutrition and Metabolism, Uppsala University, Uppsala, Sweden

**Keywords:** Epidemiology, Economics

## Abstract

Fiscal food policies can be used, among others, to minimize the burden of diet-related diseases. To inform the design of such policies in Australia, we used the large grocery-purchasing dataset NielsenIQ Homescan to estimate own-price elasticities and cross-price elasticities for 18 food categories. We found that households were most responsive to changes in price for non-sugar-sweetened beverages and sugar-sweetened beverages: a 10% increase in price was associated with reductions in demand of 15% and 12%, respectively. Additionally, an increase in the price of one category was associated with relatively small changes in the quantity demanded for other categories (that is, 92% of cross-price elasticities had an absolute value <0.2). There were small differences in own-price and cross-price elasticities across socioeconomic quintiles. These price elasticity estimates can be used to model the health and equity impacts of fiscal food policies in Australia.

## Abstract (web summary)

Food price elasticities are key to the design of policy interventions aimed at shifting food consumption. Using a comprehensive longitudinal dataset of grocery purchases, this study calculates own-price and cross-price elasticities of demand for commonly consumed food categories in Australia, both at the population level and for different socioeconomic groups.

## Main

Poor diets are linked to one in five premature deaths globally, with most of these deaths attributable to cardiovascular disease, cancer and type 2 diabetes^1^. This burden is largely attributable to excessive consumption of discretionary foods high in added salt and sugar and inadequate consumption of healthy foods (including fruits, vegetables, whole grains, nuts and seeds)^2^. To address this burden, governments around the world are adopting a range of evidence-based programmes to effectively and efficiently promote healthier dietary choices, including reformulation programmes, nutrition education programmes, marketing regulations, nutrition labelling standards and fiscal food policies^3^.

Fiscal food policies, including the use of Pigouvian taxes for unhealthy foods and subsidies for healthy foods, have gained popularity since the early 20th century^4^ as a tool to modify food prices at the point of sale, shifting consumers towards healthier diets and thereby combating the burden of diet-related diseases. Most notably, the World Health Organization (WHO) advocates for the use of taxes on sugar-sweetened beverages as a cost-effective intervention to reduce sugar consumption^5^, and to date more than 100 countries have implemented such taxes^6^. Similarly, several countries have imposed taxes on salt^7^, saturated fat^8^ and ultraprocessed foods^9^, and many provide subsidies for fruits and vegetables^10,11^. Related to these policies, there is also increasing attention on restricting price promotions for unhealthy foods and beverages^12,13^, and on calls to implement carbon taxes for certain foods to promote more environmentally sustainable diets^14^.

To design effective fiscal food policies, it is crucial to understand how changes in food price will affect the quantity of food demanded (that is, the price elasticities of demand for food). Price elasticities indicate how changing the price of a certain good is related to the quantity of it demanded (that is, own-price elasticity, OPE) and to the quantity of other goods demanded (that is, cross-price elasticities, CPEs). For food products, OPEs typically vary according to (1) the food category—staple foods (for example, rice) and foods with few substitutes (for example, salt) are relatively inelastic^15^; and (2) socioeconomic status (SES)—low-SES groups generally show relatively more elastic consumption than low-SES groups^16^. In the context of food taxes and subsidies, the stronger OPEs among low-SES groups suggest a potential for greater equity-promoting effects because these groups are likely to make greater changes to their purchasing habits.

In Australia, dietary risks are the third leading risk factor for ill-health and substantial socioeconomic disparities in diet quality are apparent^17^. However, despite calls by medical and public health bodies to introduce fiscal measures to improve diet quality^18,19^, currently there is no comprehensive set of OPEs and CPEs specific to health-related food pricing policies in Australia (for example, no CPEs for discretionary food categories)^20–22^, which may limit the ability to design effective fiscal food policies aimed at minimizing the burden of diet-related ill-health^14,23,24^. Therefore, the aim of this study was to use a large panel dataset (NielsenIQ Homescan, 2015–2019) to estimate a comprehensive set of OPEs and CPEs for 18 policy-relevant food categories in Australia. As a secondary analysis, we analysed whether these price elasticities varied according to socioeconomic level.

## Results

We obtained a sample of 16,376 households across the five years of NielsenIQ Homescan data. Of these, we excluded 6,368 because they were flagged by NielsenIQ as containing unreliable purchase information (for example, not meeting the minimum spend threshold of AU$260 per year), leaving 10,008 households. The remaining sample was unbalanced, with approximately half (*n* = 4,854) appearing in all five years of data, and the rest appearing in four years (*n* = 1,263), three years (*n* = 1,168), two years (*n* = 1,148) or one year (*n* = 1,575) of data. Households were based in Melbourne (20%), Sydney (19%), Brisbane (14%), Perth (8%), Adelaide (7%) and other areas (31%). The mean household size was 2.7 persons.

The raw data contained 40,593,062 transactions for 36,697 households over the 5-year period. These data were unbalanced because not all households recorded transactions for all 5 years. We excluded 1,164,416 transactions because they missed required product information (that is, price and/or package size), resulting in 39,428,646 transactions for 36,697 households. For each household in each year, we aggregated the purchase information according to each of the 18 categories, resulting in 660,546 observations. For each category, the proportion of positive purchases ranged from 94.4% to 99.9% (Supplementary Table 1).

The mean annual household expenditure (s.d.), adjusted to the first quarter of 2019, was AU$4730 (AU$2416). The mean annual household expenditure was slightly higher (+3%) for the highest socioeconomic quintile compared with the lowest socioeconomic quintile (Supplementary Table 2).

**Fig. 1:**
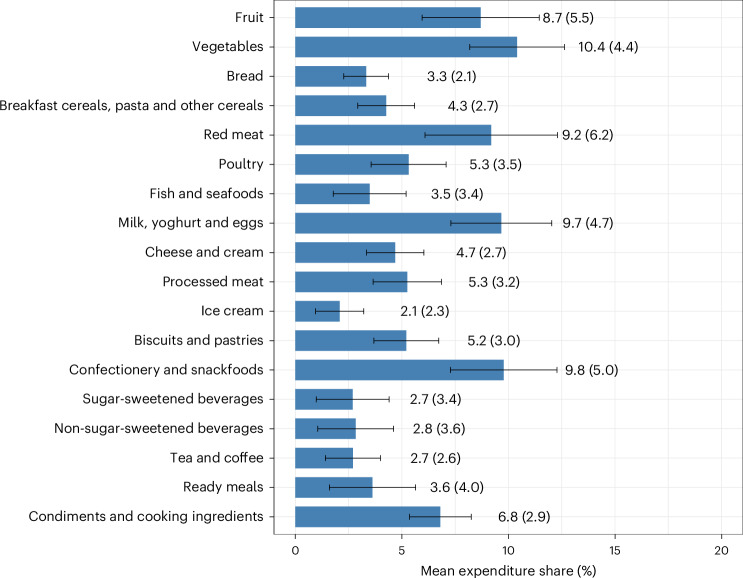
Mean annual grocery expenditure shares for Australian households in 2015–2019. Analyses were conducted using a nationally representative sample of 10,008 households in the Australian NielsenIQ Homescan dataset. Expenditure shares were calculated by dividing the annual category-level expenditure by the total annual grocery expenditure. The blue bars represent means; error bars indicate ±0.5 s.d. The numbers are means (s.d.). Expenditure shares for each calendar year and socioeconomic quintile are provided in Extended Data Figs. 1 and 2, respectively. Source data

**Extended Data Fig. 1:**
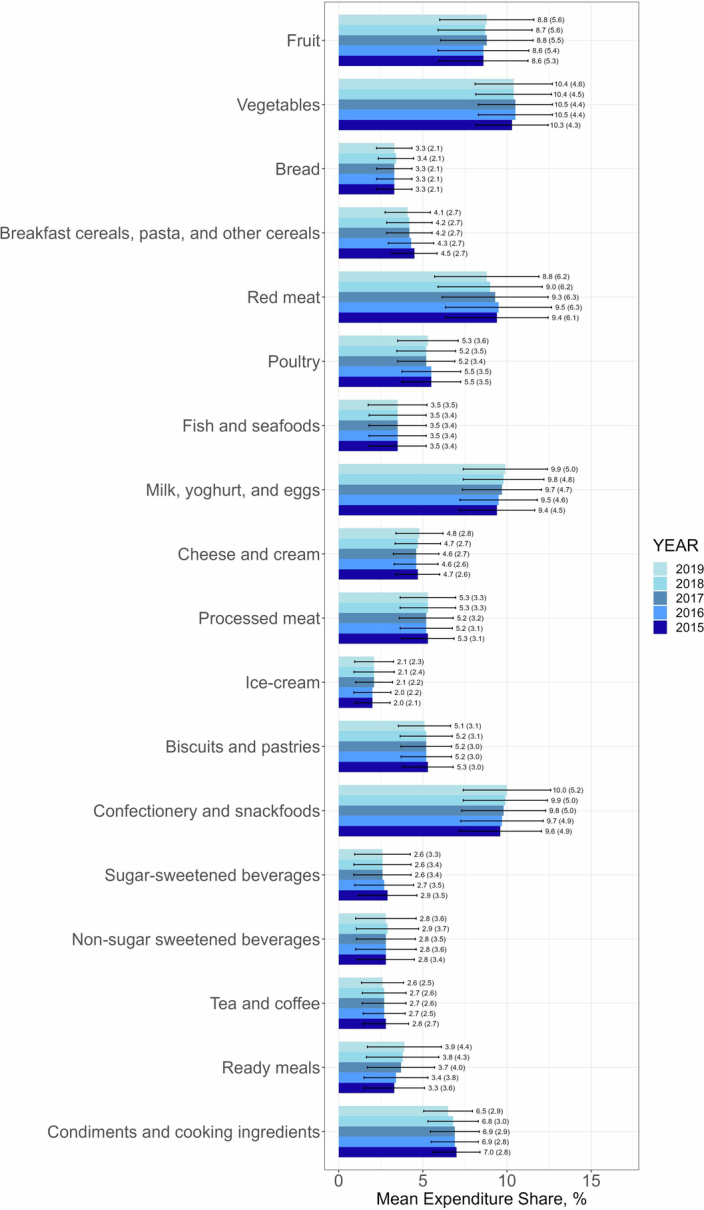
Mean annual grocery expenditure shares for Australian households by calendar year. Analyses were conducted using a nationally representative sample of 10,008 households in the Australian NielsenIQ Homescan dataset. Expenditure shares were calculated by dividing the annual category-level expenditure by the total annual grocery expenditure. The bar represents the mean value and the error bar indicates ± 0.5 standard deviations. The mean value is labelled followed by its standard deviation in parentheses.

**Extended Data Fig. 2:**
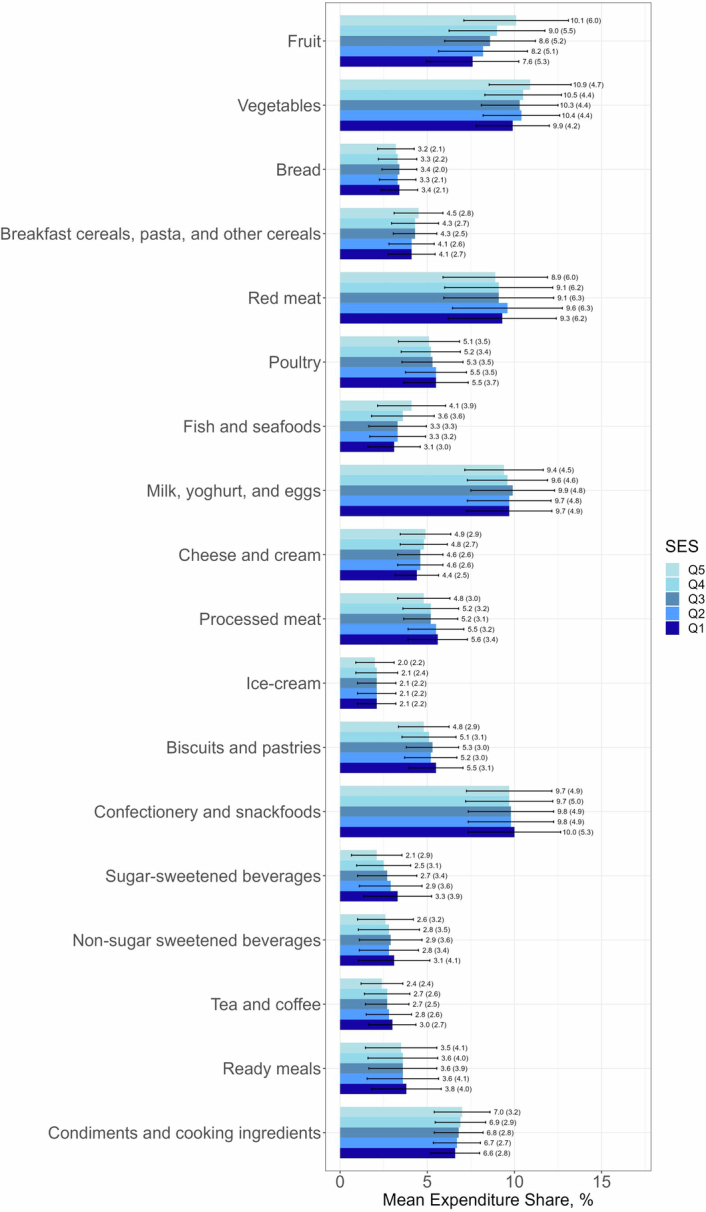
Mean annual grocery expenditure shares for Australian households by socioeconomic quintile. Analyses were conducted using a nationally representative sample of 10,008 households in the Australian NielsenIQ Homescan dataset. Expenditure shares were calculated by dividing the annual category-level expenditure by the total annual grocery expenditure. Socioeconomic quintile was defined based on the Index of Relative Social Advantage and Disadvantage, with Q1 representing the most disadvantaged areas and Q5 representing the most advantaged areas. The bar represents the mean value and the error bar indicates ± 0.5 standard deviations. The mean value is labelled followed by its standard deviation in parentheses.

As shown in Fig. 1, the mean expenditure share across the five years was highest for vegetables (10.4%) and lowest for ice cream (2.1%). For ready meals, the expenditure share grew by 18% between 2015 and 2019 (that is, from 3.3% to 3.9%), while for all other categories the expenditure share fluctuated between −9% and 7% over the study period (Extended Data Fig. 1). Compared to the lowest socioeconomic quintile, the highest socioeconomic quintile allocated a much larger proportion of their expenditure to fish and seafoods (+34%) and fruit (+33%) and a much smaller proportion to sugar-sweetened beverages (−36%) and tea and coffee (−19%) (Extended Data Fig. 2).

**Fig. 2:**
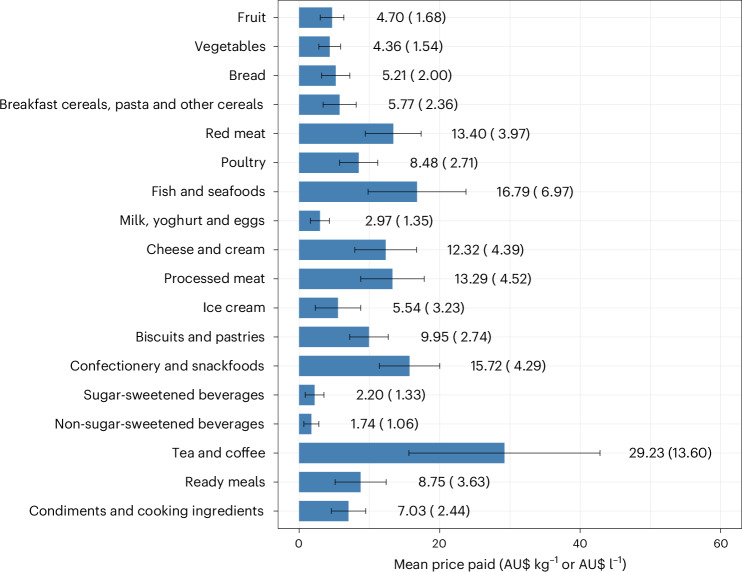
Mean food prices paid by Australian households in 2015–2019. Analyses were conducted using a nationally representative sample of 10,008 households in the Australian NielsenIQ Homescan dataset. Food price was calculated by dividing the annual category expenditure by the total annual grocery purchase quantity. Prices were adjusted for inflation to match the March 2019 quarter using the Consumer Price Index for Foods. The blue bars represent means; error bars indicate ±0.5 s.d. The numbers are means (standard deviation). Expenditure shares for each calendar year and socioeconomic quintile are provided in Extended Data Figs. 3 and 4, respectively. Source data

**Extended Data Fig. 3:**
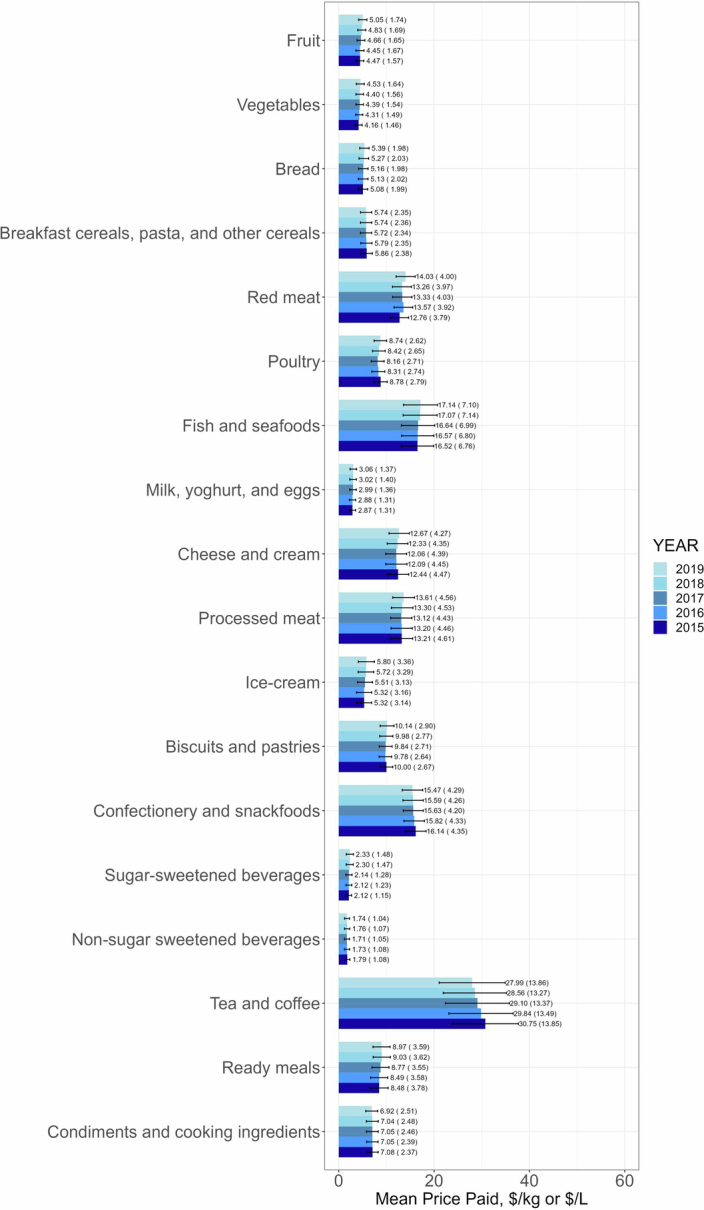
Mean food prices paid by Australian households by calendar year. Analyses were conducted using a nationally representative sample of 10,008 households in the Australian NielsenIQ Homescan dataset. Food price was calculated by dividing the annual category expenditure by the total annual grocery purchase quantity. Prices were adjusted for inflation to match the March 2019 quarter using the Consumer Price Index for Foods. The bar represents the mean value and the error bar indicates ± 0.5 standard deviations. The mean value is labelled followed by its standard deviation in parentheses.

**Extended Data Fig. 4:**
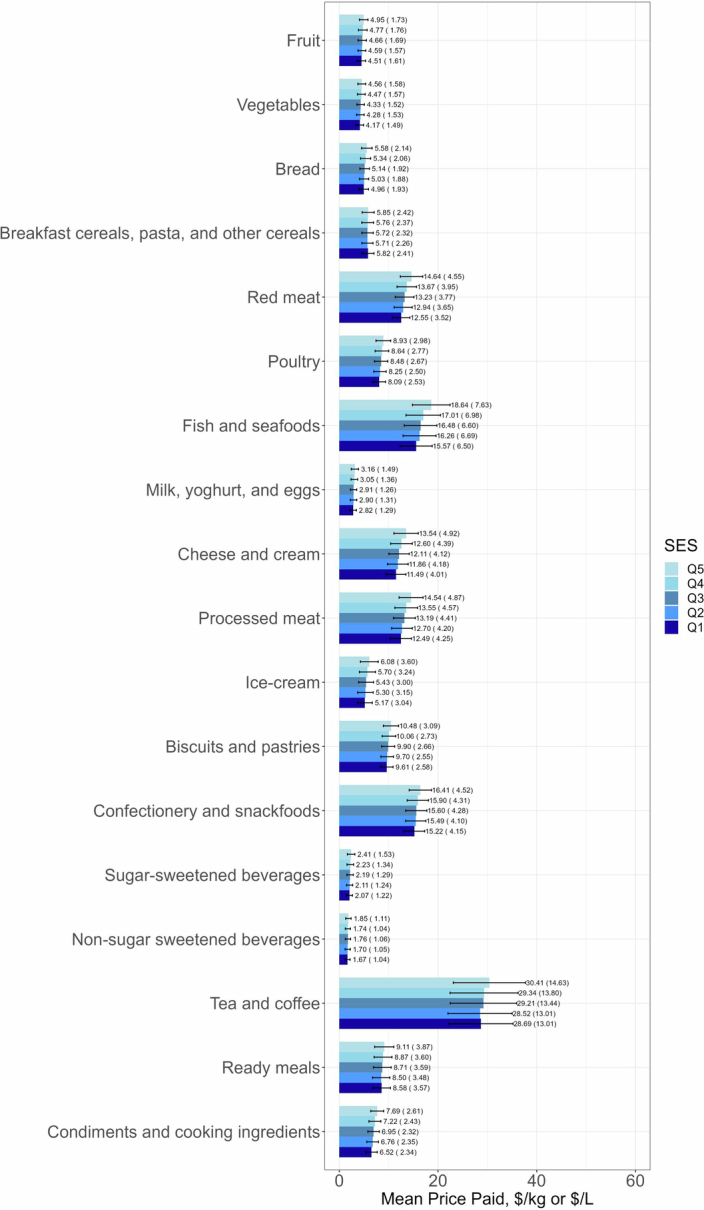
Mean food prices paid by Australian households by socioeconomic quintile. Analyses were conducted using a nationally representative sample of 10,008 households in the Australian NielsenIQ Homescan dataset. Food price was calculated by dividing the annual category expenditure by the total annual grocery purchase quantity. Prices were adjusted for inflation to match the March 2019 quarter using the Consumer Price Index for Foods. Socioeconomic quintile was defined based on the Index of Relative Social Advantage and Disadvantage, with Q1 representing the most disadvantaged areas and Q5 representing the most advantaged areas. The bar represents the mean value and the error bar indicates ± 0.5 standard deviations. The mean value is labelled followed by its standard deviation in parentheses.

The price paid varied considerably between major categories, with the mean price paid greatest for tea and coffee (AU$29.23 kg^−1^) and lowest for non-sugar-sweetened beverages (AU$1.74 l^−1^) (Fig. 2). Within food categories, the variation in price paid was substantial: the s.d. ranged from 28% to 61% of the mean value. From 2015 to 2019, the mean price paid for fruits rose by 13%, while the mean price paid for other categories changed by −9% to 10% (Extended Data Fig. 3). Additionally, the highest socioeconomic quintile paid prices that were 1–20% higher than those paid by the lowest socioeconomic quintile (Extended Data Fig. 4).

### OPEs

**Fig. 3:**
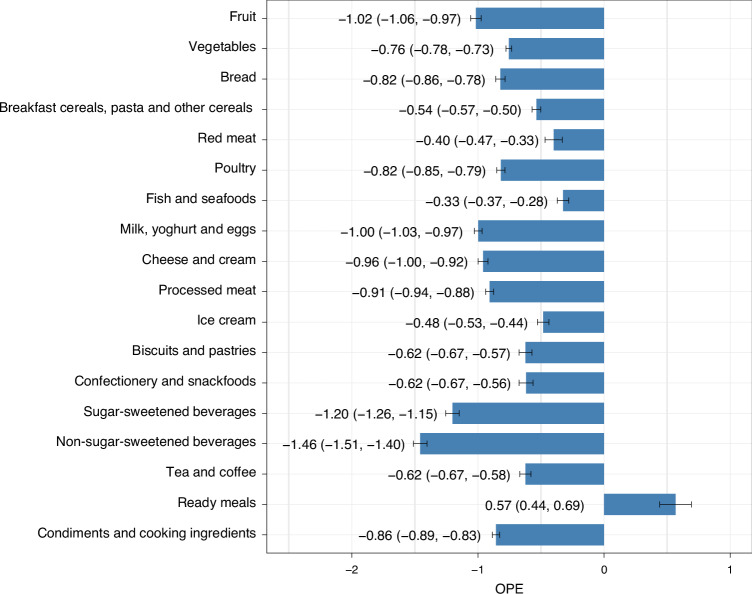
Marshallian uncompensated OPE estimates for food categories in Australia. Estimates refer to the percentage change in quantity demanded given a 1% increase in price. Estimates were produced by applying the AIDS to 660,546 price-demand observations from the Australian NielsenIQ Homescan dataset (2015–2019). The blue bars represent the point estimates of the OPEs; error bars indicate 95% confidence intervals calculated using the delta method. The numbers are the OPEs, with the corresponding 95% confidence intervals shown in parentheses. OPEs for each socioeconomic quintile are provided in Extended Data Fig. 5. Source data

**Extended Data Fig. 5:**
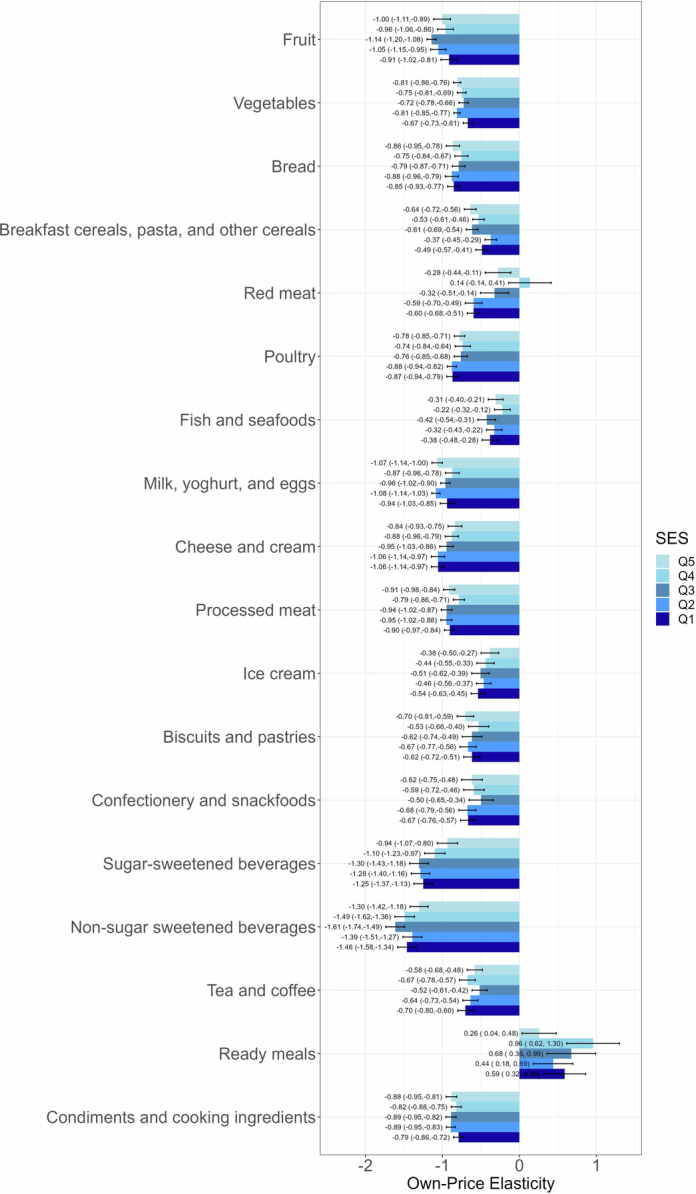
Marshallian uncompensated own-price elasticity estimates for food categories in Australia by socioeconomic quintile. Estimates refer to the percentage change in quantity demanded given a 1% increase in price. Estimates were produced by applying the Almost Ideal Demand System to 660,546 price-demand observations from the Australian NielsenIQ Homescan dataset (2015–2019), with approximately one-fifth of these observations belonging to each socioeconomic quintile. Socioeconomic quintile was defined based on the Index of Relative Social Advantage and Disadvantage, with Q1 representing the most disadvantaged areas and Q5 representing the most advantaged areas. Negative estimates indicate complements and positive estimates indicate substitutes. The bar represents the point estimate of the own-price elasticity, and the error bar indicates the 95% confidence. Source data

OPE estimates and their 95% confidence intervals are presented in Fig. 3. Households were most sensitive to changes in price for ‘non-sugar-sweetened beverages’ (OPE, −1.46) and ‘sugar-sweetened beverages’ (OPE, −1.20), indicating that a 10% increase in price was associated with a 14.6% (10% × −1.46) and 12.0% (10% × −1.20) reduction in the quantity demanded, respectively. For one category, ready meals, the OPE was positive (0.57), indicating a 10% increase in price was associated with a 5.7% increase in the quantity demanded. In general, the estimated OPEs were similar across socioeconomic quintiles: the point estimates differed by 0.01 and 0.33 between the highest and lowest socioeconomic quintiles (Extended Data Fig. 5). Considering categories of key public health significance, we found that the highest socioeconomic quintile, compared with the lowest socioeconomic quintile, was somewhat more sensitive to changes in price for vegetables (OPEs, −0.81 and −0.67, respectively), and somewhat less sensitive to changes in price for sugar-sweetened beverages (OPEs, −1.25 and −0.94, respectively). All demand systems fulfilled the conditions of additionality, symmetry and homogeneity.

### CPEs

**Fig. 4:**
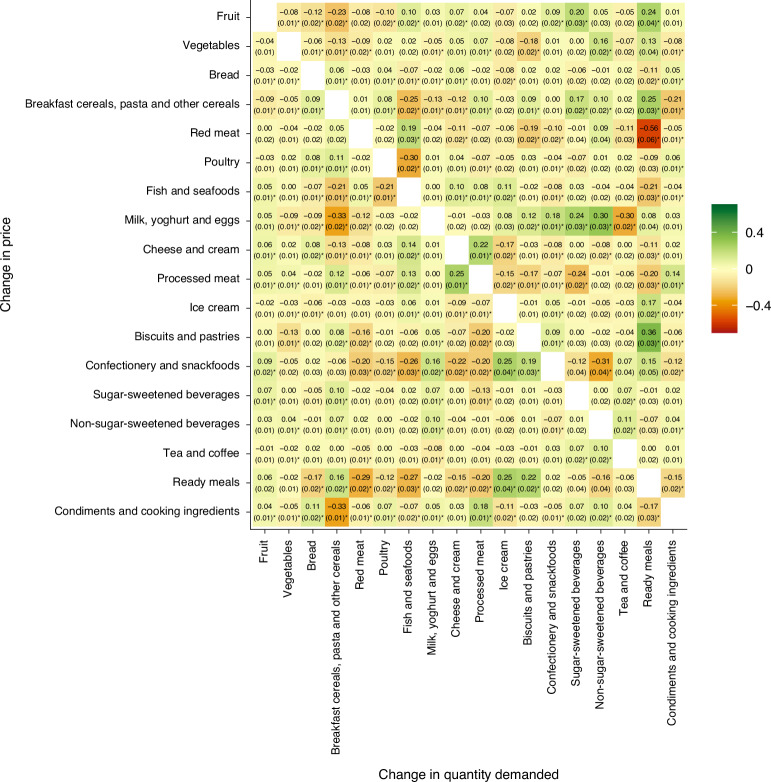
Marshallian uncompensated CPE estimates (and standard errors) for food categories in Australia. Estimates refer to the percentage change in quantity demanded for one category on the horizontal axis given a 1% increase in price in the category on the vertical axis. Estimates were produced by applying the AIDS to 660,546 price-demand observations from the Australian NielsenIQ Homescan dataset (2015–2019). Negative estimates indicate complements and positive estimates indicate substitutes. The colour scale differentiates between complements (that is, orange) and substitutes (that is, green). The standard errors in parentheses were estimated using the delta method. *Statistically significant CPEs with *P* values <0.05 from two-sided *t*-tests, with adjustments for multiple comparisons made using the Bonferroni correction. CPEs for each socioeconomic quintile are provided in Extended Data Tables 1–5.

For the 18 categories, we estimated 306 CPEs (Fig. 4). Of these, 29% were negative and statistically significant (indicating complement foods), 24% were positive and statistically significant (indicating substitutes) and 47% were not statistically significant. Only 9% (*n* = 27) had an absolute value >0.2 (indicating a 10% price increase in one category was associated with more than a 2% change in quantity demanded for another). The largest complementary relationship was found between ‘red meat’ and ‘ready meals’ (CPE, −0.56), while the largest substitutionary relationship was seen between ‘biscuits and pastries’ and ‘ready meals’ (CPE, 0.36). Considering categories of high policy interest, a 10% price increase for sugar-sweetened beverages minimally affected the demand for other categories: demand shifts were between −1.3% and 1.0%. Additionally, a 10% price increase for fruits and vegetables combined resulted in small shifts in the demand for other categories, with the largest decrease seen for ready meals (−3.7%) and the largest increase seen for breakfast cereals, pasta and other cereals (+3.6%). Furthermore, CPEs showed little variation across socioeconomic quintiles (Extended Data Tables 1–5).

### Robustness checks

Three robustness checks generated price elasticities that were very similar to the main analysis: (1) expanding the sample to include households with unreliable purchase information (for example, not meeting the minimum spend threshold of AU$260 per year), (2) using a balanced sample of households (that is, only households that were on the panel for all five years), and (3) limiting the sample to only households that made purchases from all 18 categories (Supplementary Table 3). For each of these robustness checks, at least 17 of the 18 OPE point estimates were within 0.1 units of the respective OPE point estimate from the main analysis. Additionally, a fourth robustness check that used the quadratic version of the almost ideal demand system (AIDS) produced moderately consistent price elasticities, with 10 out of 18 OPE point estimates within 0.1 units of the respective OPE point estimate from the main analysis.

## Discussion

In this study, we used a large consumer panel dataset spanning five years to estimate price elasticities for 18 food categories in Australia. We found that the OPEs were largest (in absolute value) for ‘non-sugar-sweetened beverages’ and ‘sugar-sweetened beverages’, suggesting that fiscal policies pertaining to these categories would produce the largest changes in food demand. Additionally, most food categories shared very small complementary/substitutionary relationships with other food categories, indicating that taxing/subsidizing one food category would generally be associated with very small changes in the quantity demanded for other food categories (that is, allow precision public health nutrition policies and avoid unintended consequences). There were small differences in OPEs and CPEs by socioeconomic level, which signifies that fiscal policies would probably result in proportionate changes in food demand across socioeconomic levels.

Our results provide further support for the potential health benefits of a sugar-sweetened beverage tax in Australia^20^. Importantly, we show that the substitution effects between sugar-sweetened beverages and other discretionary food categories in Australia are small. Additionally, we found that a broader tax on discretionary categories (including ‘processed meat’, ‘ice cream’, ‘biscuits and pastries’, ‘confectionery and snackfoods’ and ‘sugar-sweetened beverages’) could yield substantial public health gains, given the strong OPEs (that is, −0.48 to −1.20) and the weak CPEs (that is, −0.31 to 0.36) of these categories. Although such taxes may be slightly financially regressive, they are likely to produce large health benefits among low-SES households because these households have the highest burden of obesity-related diseases^25^. To strengthen the equity impact of these taxes, the taxation revenue could be allocated to public health initiatives for disadvantaged populations (for example, targeted fruit and vegetable subsidies^26^). However, it is important to note that the adoption of food and beverage taxes can be hindered by administrative challenges, competing national agendas, resistance from the food industry and difficulties in deciding which products to tax^27^.

Our study also suggests that fruit and vegetable subsidies could have substantial public health benefits, given the pronounced OPEs for these categories and the absence of any strong substitution effects. Interestingly, a previous Australian population modelling study^24^ that relied on New Zealand price elasticity data^28^ found that subsidizing fruits and vegetables would have a negative public health impact (because consumers allocate a large portion of savings to sugary and salty foods). Our findings suggest that it may be inappropriate to use New Zealand price elasticity data for an Australian modelling study. Future work could use epidemiological frameworks that rely on the price elasticities observed in this study to model the potential health impacts, equity impacts and cost-effectiveness of fruit and vegetable subsidies in Australia.

Our finding that OPEs were generally consistent across socioeconomic groups is consistent with a previous Australian study that conducted a discrete choice experiment and found different socioeconomic groups had small differences in OPEs for sugar-sweetened beverages^29^. Conversely, our result is inconsistent with a meta-analysis of 21 studies that found, in high-income countries, that low-SES households typically have a food price elasticity index 0.18 units greater (in absolute value) than high-SES households^16^. The reason for the apparent lack of disparity in OPEs in the Australian context is unclear. Perhaps, this is related to our result that total grocery expenditure is similar between SES groups (that is, in Australia, low-SES and high-SES households have a similar total budget for groceries and therefore respond similarly to food price changes). Additionally, it may highlight the inconsistency of income-level influence on price elasticity between populations.

Our study presents a comprehensive set of price elasticity estimates for foods and beverages in Australia. Previous studies have focused on OPEs and CPEs for beverage categories^20,29^, OPEs and CPEs for core foods^22^, and OPEs for food categories related to the goods and services tax^21^, with data spanning 1998 to 2015. Our study also estimates food price elasticities according to SES in Australia, previously restricted to OPEs for sugar-sweetened beverages^29^. Additionally, while other studies considered 1–36 months of household purchasing data, our study considered 60 months of household purchasing data, which allowed us to estimate price elasticities with a higher level of precision. The price elasticity estimates in our study may be broadly similar to those of other high-income countries with comparable food supplies and consumption patterns, in which case our results may provide guidance to policymakers and researchers in such settings as well.

Compared to the most recent Australia-specific food price elasticity study^21^, which applied the AIDS to 2013–2015 NielsenIQ data, our OPE estimates spanned a slightly larger range (that is, −1.36 to −0.41 and −1.46 to 0.57 for the previous study and our study, respectively). Considering the categories assessed in both the previous study and our study, we found similar OPE estimates for ‘biscuits and pastries’ (−0.71 versus −0.62), ‘vegetables’ (−0.82 versus −0.76) and ‘bread’ (−0.75 versus −0.82). However, our study found a notably stronger OPE estimate for ‘fruits’ (−0.68 versus −1.02) and a notably weaker OPE estimate for ‘fish and seafoods’ (−0.60 versus −0.33) and ‘breakfast cereals, pasta and other cereals’ (−0.70 versus −0.54). Because the previous study did not account for price endogeneity and frequently imputed category price information (that is, 64% of observations contained at least one category price imputation), it is possible the price elasticity estimates in our study are more robust. Alternatively, these differences may be due to differences in demand model specifications (for example, selection of competitor food categories) and/or temporal trends in consumer preferences^30^.

Our study had some limitations. First, the accuracy of the price and purchase quantity information in the Australian NielsenIQ Homescan dataset has not been reported; however, analysis of a US NielsenIQ Homescan shows similar accuracy to other economic surveys^31^. Second, although we minimized price endogeneity using Fisher Ideal Price indexes, an even more robust approach would have been to use market value price inputs, as has been done in other studies^28^. However, market value price inputs provided by the Australian Bureau of Statistics^32^ are limited to a narrow range of food categories, and therefore our approach allowed for more comprehensive food price elasticity estimation. Third, in the process of assessing 18 food categories, we aggregated some nutritionally diverse foods within a single food category (for example, milk, yoghurt and eggs), thereby limiting the ability to see price elasticities for more specific categories. Fourth, we found a positive association between price and demand for ready meals, which does not align with the law of demand. Ready meals showed a steep increase in popularity over the study period^33^, suggesting it is possible that consumers’ growing preference for convenience confounded the results, and therefore the results for this category should be interpreted with caution. Finally, it is worth noting a recent meta-analysis of 95 food price elasticity studies that found OPE estimates produced using scanner data tend to be greater in absolute value than estimates produced using other surveys (for example, household consumption surveys and market data), although these authors comment there is no way to ascertain which data tend to produce the most accurate price elasticities^34^.

Our results demonstrate the potential for fiscal food policies to shift dietary patterns in Australia. These price elasticity estimates will be critical for designing cost-effective fiscal food policies aimed at minimizing the burden of diet-related ill-health in Australia. Importantly, many policy-relevant categories exhibited high price sensitivity and did not share strong a cross-relationship with other categories, demonstrating the potential to tax/subsidize these categories and produce large changes in population food intakes with minimal substitution effects.

## Methods

### Food demand dataset

We used five years of household food purchasing data in the Australian NielsenIQ Homescan Dataset (that is, January 2015–December 2019^35^). This longitudinal dataset comprises a panel of approximately 10,000 households in each year who recorded all packaged and unpackaged foods purchased for at-home consumption (that is, from supermarkets, convenience stores and grocers). The panel is broadly representative of the Australian population in terms of geographic distribution, income levels and household sizes^36^. The sample of households is unbalanced because participation on the panel varied between one and five years. To collect the data, households used a portable barcode scanner to scan product barcodes (for packaged items) and a scanning guide booklet (for unpackaged items such as fresh fruits and vegetables). For each scanned product, panel members manually recorded the price paid and the quantity purchased. NielsenIQ then determined each product’s name, brand, package size (kg or litres) and food category via linkage with a master product dataset. No data were collected on products purchased for consumption outside of the home (for example, from restaurants and cafés).

Households that were flagged by NielsenIQ for containing unreliable purchase information were excluded from the study. For each calendar year, households were flagged if they (1) were not on the panel for the entire 52-week period, (2) did not scan a barcode for at least 26 weeks or (3) did not meet the minimum spend threshold (that is, an average of AU$5 per week).

To account for inflation over the study period, we chose January–March 2019 as the base quarter and adjusted prices in other quarters using the Consumer Price Index for foods^37^. Additionally, we flagged and excluded all purchases with implausible prices: (1) more than 5 s.d. above the category-level unit value, or (2) more than 5 s.d. above the brand-level unit value within the category.

### SES classification

Household SES was categorized using the Index of Relative Social Advantage and Disadvantage (IRSAD) as defined by the Australian Bureau of Statistics^38^. This index summarizes the economic and social conditions of households living within a particular area by considering a range of indicators, such as income levels, education levels, employment rates and housing. To maintain household confidentiality, NielsenIQ does not provide the exact IRSAD score of each household, but instead aggregates IRSAD scores into deciles. Using these scores, we classified households into socioeconomic quintiles (that is, Q1 referred to IRSAD deciles 1 and 2 and Q5 referred to IRSAD deciles 9 and 10).

### Category selection

Products were organized into 18 different categories (Supplementary Table 4). The categorization system was adapted from the categorization system used for the WHO Nutrient Profile Model for the Western Pacific and South East Asia Regions^39,40^. Given that these Nutrient Profile Models were primarily developed to support policies restricting marketing of unhealthy foods towards children, rather than to provide guidance for fiscal policies, we made some adaptations to better align with plausible fiscal policies in Australia. For example, we disaggregated the beverages category into sugar-sweetened beverages, non-sugar-sweetened beverages, and tea and coffee, and also divided the meat category into red meat, poultry, and fish and seafoods. Definitions of each category are provided in Supplementary Table 5. Each category was further classified as ‘core’, ‘discretionary’ or ‘other’ according to the Australian Dietary Guidelines^41^.

### Empirical approach

We used the linearized version of the AIDS developed by Deaton and Muellbauer^42^ to estimate food price elasticities. This approach has previously been applied to Australian NielsenIQ data to estimate a subset of food price elasticities^20,21^. In brief, we aggregated transactions annually for each household. Then, the expenditure share of each category (that is, category expenditure divided by total expenditure) was modelled as a function of its price, the price of other categories and the total expenditure.

#### Estimating category prices

To estimate category prices, one option was to use unit values (that is, category expenditure divided by purchase quantity) for each household in each year. However, unit values may introduce endogeneity into the model because household preferences for quality (omitted variable) can influence both unit values (independent variables) and category expenditure shares (dependent variables). Based on Zhen et al.^43^ and Sharma et al.^20^, to reduce this endogeneity bias, we represented category prices using a Fisher Ideal Price Index based on brand-level unit values and quantities, as shown in equation (1):

1 $${p}_{{jht}}=\sqrt{\frac{{\sum }_{k=1}^{n}{p}_{{kht}}{q}_{k0}}{{\sum }_{k=1}^{n}{p}_{k0}{q}_{k0}}\times \frac{{\sum }_{k=1}^{n}{p}_{{kht}}{q}_{{kht}}}{{\sum }_{k=1}^{n}{p}_{k0}{q}_{{kht}}}}$$

Here, *p*_*jht*_ is the Fisher Ideal Price Index for the *j*th category for household *h* in year *t*, *p*_*kht*_ is the unit value of brand *k* for household *h* in year *t*, *q*_*kht*_ is the purchase quantity of brand *k* for household *h* in year *t*, *p*_*k*0_ is the mean unit value of the *k*th brand across all years and households, *q*_*k*0_ is the mean purchase quantity of the *k*th brand across all years and households, and *n* is the number of brands.

Wherever households did not purchase a given brand, it was not possible to calculate the corresponding brand unit value. Here, we imputed the brand-level unit value using regression and available brand prices, household size, year and region information, as has been done in prior studies^20,43^. Additionally, for each category, to reduce the number of imputations, we only considered the top brands that accounted for 80% of sales revenue and combined all other brands into a single group.

#### Estimating total expenditure

For each household in each year, total expenditure was calculated by summing all food and beverage purchases. However, using total expenditure as an independent variable in the model may introduce endogeneity because it is co-determined with category expenditure shares, thereby representing a simultaneity bias^43^. To mitigate this bias, we adapted a commonly used approach in the literature^20,44^ and used the natural logarithm of household annual income divided by the Stone Price Index as an instrumental variable, as shown in equations (2) and (3):

2 $$\mathrm{ln}\left(\frac{{{\rm{X}}}_{{ht}}}{{P}_{{ht}}}\right)={\lambda }_{1}+{\lambda }_{2}\mathrm{ln}\left(\frac{{I}_{{ht}}}{{P}_{{ht}}}\right)+{\lambda }_{3}\mathrm{ln}\left({H}_{{ht}}\right)+{\tau }_{t}+{R}_{r}+{\nu }_{{ht}}$$

3 $$\mathrm{ln}\left({P}_{{ht}}\right)=\mathop{\sum }\limits_{j=1}^{m}{w}_{j{ht}}\mathrm{ln}\left({p}_{j{ht}}\right)$$

Here, *X*_*ht*_ is the total expenditure of household *h* in year *t*, *P*_*ht*_ is the Stone Price Index for household *h* in year *t*, *w*_*jht*_ is the expenditure share of the *j*th major category for household *h* in year *t*, *I*_*ht*_ is the annual income of household *h* in year *t*, *H*_*ht*_ is the size of household *h* in year *t*, *τ*_*t*_ is time fixed effects, *R* is region fixed effects, and *ν*_*ht*_ is the error term.

#### Demand system

After accounting for price and expenditure endogeneity, the AIDS was estimated using equation (4) below. As part of this demand system, we imposed assumptions that made the demand system consistent with consumer choice, including additionality, symmetry and homogeneity.

4 $${w}_{{iht}}={\alpha }_{i}+\mathop{\sum }\limits_{j=1}^{m}{\gamma }_{{ij}}\mathrm{ln}\left({p}_{{jht}}\right)+{\beta }_{i}\mathrm{ln}\widehat{\left(\frac{{X}_{{\rm{ht}}}}{{P}_{{ht}}}\right)}{\delta }_{i}\ {H}_{{ht}}+{\tau }_{t}+{R}_{r}+{\mu }_{{iht}}$$

Here, $\mathrm{ln}\widehat{\left(\frac{{X}_{{\rm{ht}}}}{{P}_{{ht}}}\right)}$ was predicted using equation (2) and µ_*iht*_ is the error term. Fixed effects were used for household size, year and region. The key parameters to estimate included *γ*_*ij*_ and *β*_*i*_. One expenditure share was dropped during estimation to avoid perfect multicollinearity.

To estimate the Marshallian (uncompensated) price elasticity of major category *i* in response to price changes for major category *j* (*e*_*ij*_), we used equation (5):

5 $${e}_{{ij}}=\frac{{\gamma }_{{ij}}-{\beta }_{i}{\overline{w}}_{j}}{\overline{{w}_{i}}}-{\delta }_{{ij}}$$

In these equations, $\overline{{w}_{i}}$ and $\overline{{w}_{j}}$ are the mean expenditure share of the *i*th and *j*th category, respectively, and *δ*_*ij*_ is the Kronecker delta (that is, *δ*_*ij*_ = 1 for OPEs where *i* = *j* and *δ*_*ij*_ = 0 for CPEs). The delta method was used to estimate standard errors for price elasticities. A two-sided *t*-test was applied to each price elasticity estimate, and statistical significance was defined as a *P-*value <0.05 while adjusting for multiple comparisons using the Bonferroni correction.

Furthermore, to examine price responsiveness for each socioeconomic quintile, a demand system was estimated separately for each socioeconomic quintile. Throughout, analyses were conducted using R and the micEconAids library.

#### Robustness checks

Additionally, as a robustness check, we reconducted the empirical approach using three separate inclusion criteria: (1) including households that were flagged by NielsenIQ for containing unreliable purchase information, (2) using a balanced sample (that is, only households that were on the panel across all five years), and (3) limiting to households that made purchases from all 18 categories each year.

We also repeated the analysis using the quadratic version of AIDS, which is a more flexible model that incorporates a quadratic expenditure-share food Engel curve. Because the micEconAids library does not directly support the quadratic version of AIDS, we followed a previous study^45^ and incorporated the quadratic term as a demand shifter, as shown in equation (6):

6 $$\begin{array}{l}{w}_{i{ht}}={\alpha }_{i}+{\sum }_{j=1}^{m}{\gamma }_{{ij}}\mathrm{ln}\left({p}_{j{ht}}\right)+{\beta }_{i}\mathrm{ln}\widehat{\left(\frac{{X}_{{\rm{ht}}}}{{P}_{{ht}}}\right)}+\\{\theta }_{i}\frac{{\left(\mathrm{ln}\widehat{\left(\frac{{X}_{{\rm{ht}}}}{{P}_{{ht}}}\right)}\right)}^{2}}{{Q}_{{ht}}}+{\delta }_{i}\ {H}_{{ht}}+{\tau }_{t}\,+{R}_{r}+{\mu }_{i{ht}}\end{array}$$

Here, ${(\mathrm{ln}\widehat{(\frac{{X}_{{\rm{ht}}}}{{P}_{{ht}}})})}^{2}$ was predicted using $(\mathrm{ln}(\frac{{I}_{{\rm{ht}}}}{{P}_{{ht}}}))$ and ${(\mathrm{ln}(\frac{{I}_{{\rm{ht}}}}{{P}_{{ht}}}))}^{2}$ as instrumental variables and *Q* is the Cobb–Douglas aggregate price defined using ${Q}_{{ht}}={\prod }_{j=1}^{m}{\left({p}_{{jht}}\right)}^{{\beta }_{i}}$ (where *β*_*i*_ was estimated using equation (4)).

### Reporting summary

Further information on research design is available in the Nature Portfolio Reporting Summary linked to this article.

## Supplementary information

Supplementary Information Supplementary Tables 1–5.

Reporting Summary

## Source data

Source Data Figs. 1–3, Extended Data Fig. 5 and Extended Data Tables 1–5 Statistical source data.

## Figures and Tables

**Extended Data Table 1:**
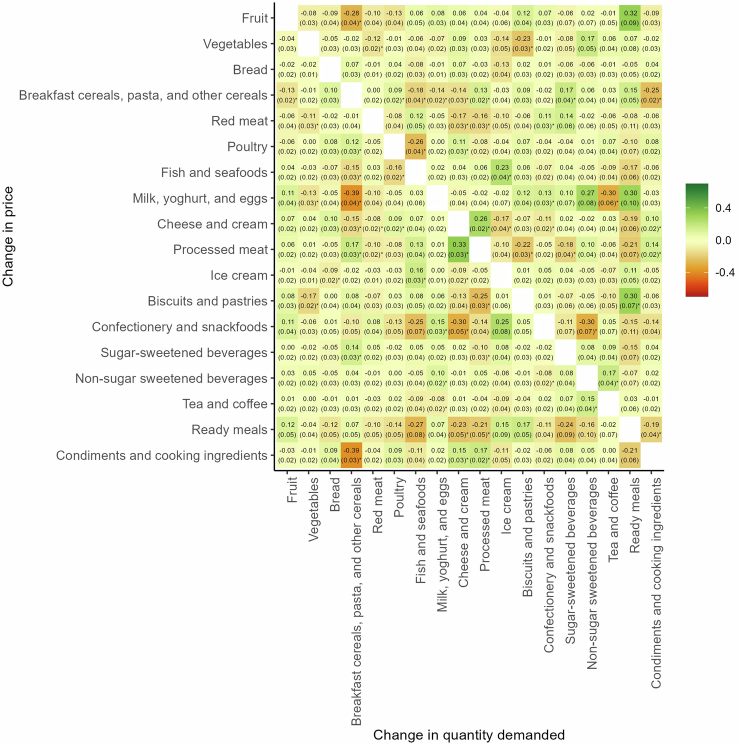
Marshallian uncompensated cross-price elasticity estimates (and standard errors) for food categories in Australia for the first socioeconomic quintile

Estimates refer to the percentage change in quantity demanded for one category on the horizontal axis given a 1% increase in price in the category on the vertical axis. Estimates were produced by applying the Almost Ideal Demand System to 133,020 price-demand observations from the Australian NielsenIQ Homescan dataset (2015–2019). Socioeconomic quintile was defined based on the Index of Relative Social Advantage and Disadvantage, with Q1 representing the most disadvantaged areas and Q5 representing the most advantaged areas. Negative estimates indicate complements and positive estimates indicate substitutes. The colour scale differentiates between complements (that is, orange) and substitutes (that is, green). The standard errors in parentheses were estimated using the delta method. Statistically significant cross-price elasticities are denoted by an asterisk (*), indicating *p* values < 0.05 from two-sided t-tests, with adjustments for multiple comparisons made using the Bonferroni correction.

Source data

**Extended Data Table 2:**
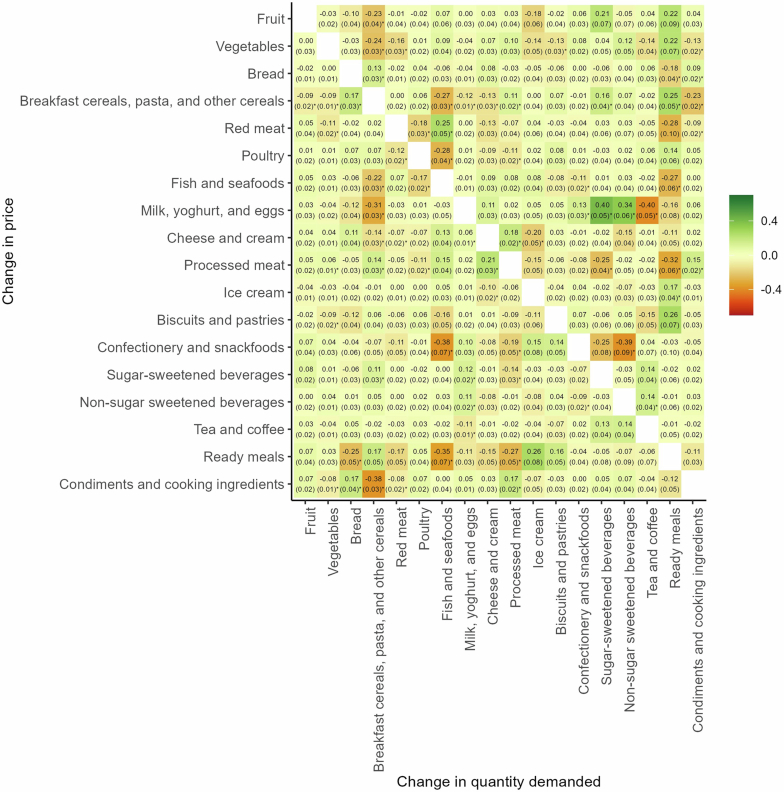
Marshallian uncompensated cross-price elasticity estimates (and standard errors) for food categories in Australia for the second socioeconomic quintile

Estimates refer to the percentage change in quantity demanded for one category on the horizontal axis given a 1% increase in price in the category on the vertical axis. Estimates were produced by applying the Almost Ideal Demand System to 132,894 price-demand observations from the Australian NielsenIQ Homescan dataset (2015–2019). Socioeconomic quintile was defined based on the Index of Relative Social Advantage and Disadvantage, with Q1 representing the most disadvantaged areas and Q5 representing the most advantaged areas. Negative estimates indicate complements and positive estimates indicate substitutes. The colour scale differentiates between complements (that is, orange) and substitutes (that is, green). The standard errors in parentheses were estimated using the delta method. Statistically significant cross-price elasticities are denoted by an asterisk (*), indicating *p* values < 0.05 from two-sided t-tests, with adjustments for multiple comparisons made using the Bonferroni correction.

Source data

**Extended Data Table 3:**
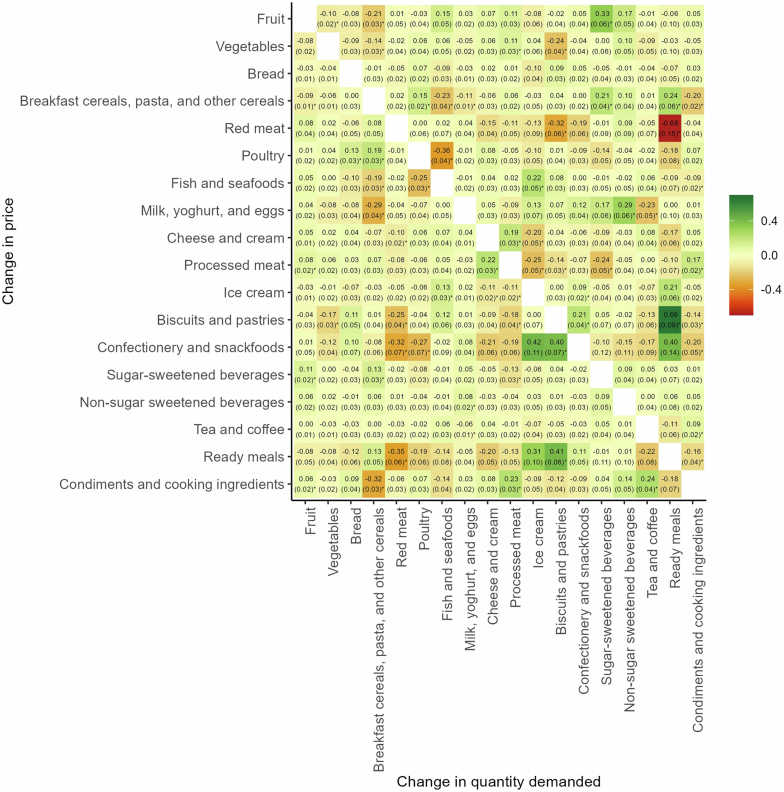
Marshallian uncompensated cross-price elasticity estimates (and standard errors) for food categories in Australia for the third socioeconomic quintile

Estimates refer to the percentage change in quantity demanded for one category on the horizontal axis given a 1% increase in price in the category on the vertical axis. Estimates were produced by applying the Almost Ideal Demand System to 127,800 price-demand observations from the Australian NielsenIQ Homescan dataset (2015–2019). Socioeconomic quintile was defined based on the Index of Relative Social Advantage and Disadvantage, with Q1 representing the most disadvantaged areas and Q5 representing the most advantaged areas. Negative estimates indicate complements and positive estimates indicate substitutes. The colour scale differentiates between complements (that is, orange) and substitutes (that is, green). The standard errors in parentheses were estimated using the delta method. Statistically significant cross-price elasticities are denoted by an asterisk (*), indicating *p* values < 0.05 from two-sided t-tests, with adjustments for multiple comparisons made using the Bonferroni correction.

Source data

**Extended Data Table 4:**
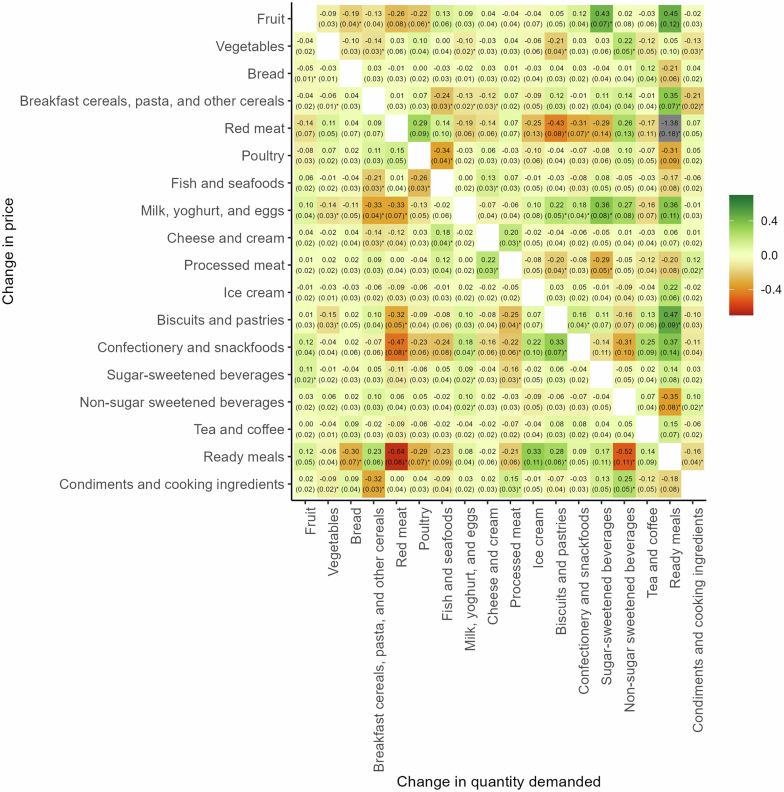
Marshallian uncompensated cross-price elasticity estimates (and standard errors) for food categories in Australia for the fourth socioeconomic quintile

Estimates refer to the percentage change in quantity demanded for one category on the horizontal axis given a 1% increase in price in the category on the vertical axis. Estimates were produced by applying the Almost Ideal Demand System to 137,412 price-demand observations from the Australian NielsenIQ Homescan dataset (2015–2019). Socioeconomic quintile was defined based on the Index of Relative Social Advantage and Disadvantage, with Q1 representing the most disadvantaged areas and Q5 representing the most advantaged areas. Negative estimates indicate complements and positive estimates indicate substitutes. The colour scale differentiates between complements (that is, orange) and substitutes (that is, green). The standard errors in parentheses were estimated using the delta method. Statistically significant cross-price elasticities are denoted by an asterisk (*), indicating *p* values < 0.05 from two-sided t-tests, with adjustments for multiple comparisons made using the Bonferroni correction.

Source data

**Extended Data Table 5:**
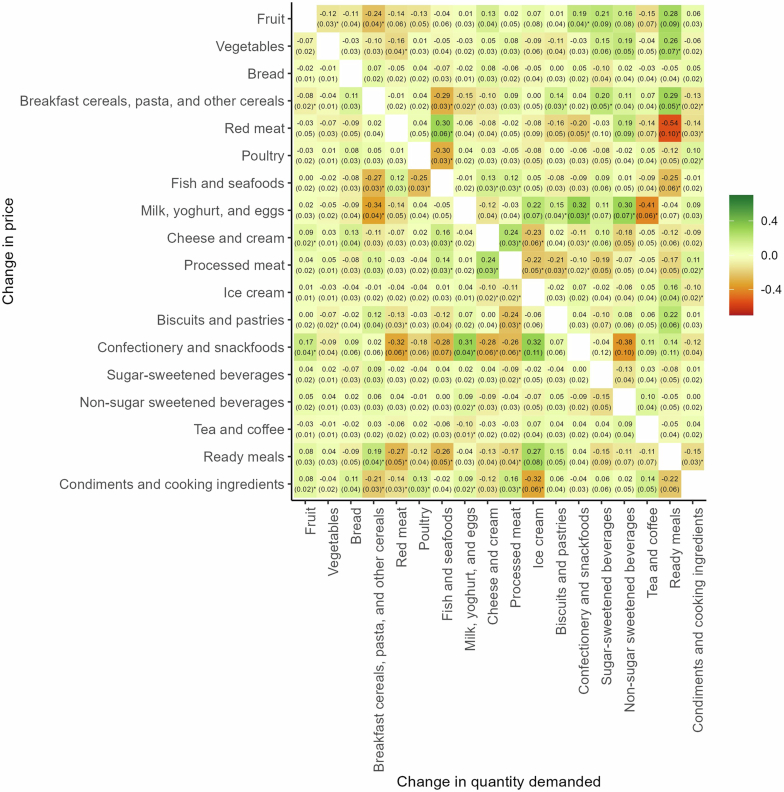
Marshallian uncompensated cross-price elasticity estimates (and standard errors) for food categories in Australia for the fifth socioeconomic quintile

Estimates refer to the percentage change in quantity demanded for one category on the horizontal axis given a 1% increase in price in the category on the vertical axis. Estimates were produced by applying the Almost Ideal Demand System to 129,420 price-demand observations from the Australian NielsenIQ Homescan dataset (2015–2019). Socioeconomic quintile was defined based on the Index of Relative Social Advantage and Disadvantage, with Q1 representing the most disadvantaged areas and Q5 representing the most advantaged areas. Negative estimates indicate complements and positive estimates indicate substitutes. The colour scale differentiates between complements (that is, orange) and substitutes (that is, green). The standard errors in parentheses were estimated using the delta method. Statistically significant cross-price elasticities are denoted by an asterisk (*), indicating *p* values < 0.05 from two-sided t-tests, with adjustments for multiple comparisons made using the Bonferroni correction.

Source data

## Data Availability

The data supporting the findings of this study are proprietary to NielsenIQ and cannot be shared publicly. Interested parties seeking access to the NielsenIQ Homescan dataset are encouraged to contact NielsenIQ directly through their official website. Source data are provided with this paper.

## Code availability

The code used to generate price elasticities is available via https://github.com/tazmandavies/Food-price-elasticity.
