# Supplementary material for: Food price elasticity estimates in Australia
Source: Nat Food. 2025 Jul 11;6(7):725–32. doi: 10.1038/s43016-025-01184-1 (PMC12283402; doi:10.1038/s43016-025-01184-1)
Supplement: Supplementary file 1 — Supplementary Tables 1–5. [file 43016_2025_1184_MOESM1_ESM.pdf]

nature food

Supplementary information https://doi.org/10.1038/s43016-025-01184-1

Food price elasticity estimates in Australia

In the format provided by the authors and unedited

Supplementary Table 1: Proportion of households with annual purchases for each food category in the 2015 – 2019 NielsenIQ Homescan dataset

Category	Proportion of purchasing households
Fruit	99.9
Vegetables	99.9
Bread	99.2
Breakfast cereals, pasta, and other cereals	99.8
Red meat	97.1
Poultry	97.1
Fish and seafoods	95.3
Milk, yoghurt, and eggs	99.9
Cheese and cream	99.4
Processed meat	99.2
Ice cream	94.4
Biscuits and pastries	99.7
Confectionery and snackfoods	99.8
Sugar-sweetened beverages	95.3
Non-sugar sweetened beverages	95.5
Tea and coffee	96.4
Ready meals	99.1
Condiments and cooking ingredients	99.9

Supplementary Table 2: Total expenditure by socioeconomic quintile and year

	Total annual household expenditure (AU$) *	
	**Mean**	**Standard deviation**
SES		
Q5	4822	2540
Q4	4736	2413
Q3	4776	2453
Q2	4650	2294
Q1	4672	2374
Year		
2019	4678	2412
2018	4692	2413
2017	4708	2402
2016	4853	2462
2015	4724	2386
Population	4731	2416

*To account for inflation over the study period, we chose January – March 2019 as the base quarter and adjusted prices in other quarters using the Consumer Price Index for Foods

Supplementary Table 3: Marshallian uncompensated own-price elasticity estimates (and 95% confidence intervals) using different samples and model specifications

	Main analysis (n = 660,546 observations)	Including households with unreliable purchase info (n = 1,019,106 observations)	Balanced sample of households (n = 436,860 observations)	Households that purchased from all 18 categories (n = 513,270 observations)	QUAIDS* (n = 660,546 observations)
Fruit	-1.02 (-1.06, -0.97)	-0.98 (-1.03, -0.94)	-1.00 (-1.06, -0.95)	-0.93 (-1.00, -0.86)	-0.36 (-0.74, 0.02)
Vegetables	-0.76 (-0.78, -0.73)	-0.78 (-0.80, -0.76)	-0.75 (-0.77, -0.72)	-0.74 (-0.76, -0.72)	-0.60 (-0.71, -0.50)
Bread	-0.82 (-0.86, -0.78)	-0.82 (-0.86, -0.77)	-0.84 (-0.88, -0.79)	-0.82 (-0.86, -0.78)	-0.82 (-0.90, -0.74)
Breakfast cereals, pasta, and other cereals	-0.54 (-0.57, -0.50)	-0.60 (-0.64, -0.56)	-0.51 (-0.56, -0.47)	-0.48 (-0.52, -0.44)	-0.34 (-0.41, -0.28)
Red meat	-0.40 (-0.47, -0.33)	-0.46 (-0.52, -0.40)	-0.36 (-0.44, -0.27)	-0.40 (-0.48, -0.31)	-0.34 (-0.59, -0.09)
Poultry	-0.82 (-0.85, -0.79)	-0.83 (-0.86, -0.79)	-0.82 (-0.86, -0.78)	-0.83 (-0.86, -0.79)	-0.77 (-0.86, -0.67)
Fish and seafoods	-0.33 (-0.37, -0.28)	-0.29 (-0.34, -0.24)	-0.31 (-0.36, -0.25)	-0.34 (-0.39, -0.29)	-0.26 (-0.36, -0.16)
Milk, yoghurt, and eggs	-1.00 (-1.03, -0.97)	-1.02 (-1.05, -0.99)	-1.00 (-1.04, -0.96)	-0.98 (-1.01, -0.95)	-0.04 (-0.54, 0.46)
Cheese and cream	-0.96 (-1.00, -0.92)	-0.97 (-1.02, -0.92)	-0.88 (-0.93, -0.84)	-0.89 (-0.94, -0.85)	-1.04 (-1.08, -1.01)
Processed meat	-0.91 (-0.94, -0.88)	-0.91 (-0.95, -0.87)	-0.87 (-0.91, -0.83)	-0.86 (-0.90, -0.83)	-0.76 (-0.85, -0.67)
Ice cream	-0.48 (-0.53, -0.44)	-0.50 (-0.55, -0.45)	-0.47 (-0.52, -0.41)	-0.48 (-0.53, -0.43)	-0.49 (-0.55, -0.43)
Biscuits and pastries	-0.62 (-0.67, -0.57)	-0.72 (-0.77, -0.67)	-0.62 (-0.68, -0.56)	-0.60 (-0.66, -0.54)	-0.69 (-0.76, -0.62)
Confectionery and snackfoods	-0.62 (-0.67, -0.56)	-0.66 (-0.72, -0.61)	-0.65 (-0.72, -0.59)	-0.65 (-0.71, -0.59)	-0.43 (-0.57, -0.29)
Sugar-sweetened beverages	-1.20 (-1.26, -1.15)	-1.16 (-1.23, -1.10)	-1.18 (-1.24, -1.12)	-1.21 (-1.26, -1.15)	-0.47 (-0.69, -0.25)
Non-sugar sweetened beverages	-1.46 (-1.51, -1.40)	-1.45 (-1.51, -1.39)	-1.45 (-1.52, -1.39)	-1.42 (-1.48, -1.36)	-1.34 (-1.44, -1.25)
Tea and coffee	-0.62 (-0.67, -0.58)	-0.61 (-0.67, -0.56)	-0.63 (-0.69, -0.58)	-0.64 (-0.69, -0.59)	-0.67 (-0.71, -0.63)
Ready meals	0.57 (0.44, 0.69)	0.36 (0.25, 0.47)	0.55 (0.40, 0.70)	0.27 (0.15, 0.39)	0.52 (0.28, 0.76)
Condiments and cooking ingredients	-0.86 (-0.89, -0.83)	-0.86 (-0.89, -0.82)	-0.84 (-0.88, -0.81)	-0.87 (-0.90, -0.84)	-0.33 (-0.51, -0.16)

QUAIDS; quadratic version of the Almost Ideal Demand System

*The QUAIDS model was not able to solve for 18 categories given the high computational demand. Therefore, the model was conducted in two parts: (i) all core categories (n = 9) and (ii) all discretionary and other categories (n = 9). For each run, the expenditure shares, total expenditure, stone price index, and equations were recalculated according to the selection of categories.

Supplementary Table 4: Food categories and alignment with Australian dietary guidelines

Group	Considered categories
Core	(1) Fruit
	(2) Vegetables
	(3) Bread
	(4) Breakfast cereals, pasta, and other cereals
	(5) Red meat
	(6) Poultry
	(7) Fish and seafoods
	(8) Milk, yoghurt, and eggs
	(9) Cheese and cream
Discretionary	(10) Processed meat
	(11) Ice cream
	(12) Biscuits and pastries
	(13) Confectionery and snackfoods
	(14) Sugar-sweetened beverages
Other	(15) Non-sugar sweetened beverages
	(16) Tea and coffee
	(17) Ready meals
	(18) Condiments and cooking ingredients

Supplementary Table 5: Category definitions

Category	Included products
**Fruit**	Frozen fruit, fresh fruit, canned fruit, dried fruit
**Vegetables**	Frozen vegetables, fresh vegetables, canned vegetables, dried vegetables, beans, peas, lentils
**Bread**	White bread, wholemeal bread, flat bread, mixed grain bread
**Breakfast cereals, pasta, and other cereals**	Muesli, ready-to-eat cereal, brans, sweet cereals, rice, pasta, noodles, flour, baking needs, spaghetti, cereal/muesli bars
**Red meat**	Beef, pork, lamb, veal, kangaroo
**Poultry**	Chicken, turkey, duck
**Fish and seafoods**	Fresh seafood, frozen seafood, chilled seafood
**Milk, yoghurt, and eggs**	Fresh milk, long-life milk, flavoured milk, milk alternatives, yoghurt, yoghurt drinks, custard, eggs
**Cheese and cream**	Hard cheese, processed cheese, soft cheese, cream
**Processed meat**	Sausages, bacon, ham, canned meats, pastries containing meat
**Ice cream**	Ice-cream, edible ices
**Biscuits and pastries**	Sweet biscuits, savoury biscuits, cakes, muffins, pastries, desserts, mixes and batters
**Confectionery and snackfoods**	Chocolate bars, Easter/Christmas chocolates, cooking chocolates, sugar, confectionery, sugar confectionery, gelatine treats, throat soothers, crisps, snackfoods, nuts, fruit bars
**Sugar-sweetened beverages**	Soft drinks with added sugar, fruit/vegetable juices with added sugar, cordials
**Non-sugar sweetened beverages**	100% fruit/vegetable juices, soda water, water, mineral waters
**Tea and coffee**	Coffee, tea, coffee substitutes, milk additive (e.g., Milo)
**Ready meals**	Frozen meals, meal kits, frozen pizza, soup, baked beans, vegetable proteins
**Condiments and cooking ingredients**	Butter, margarine, oils and fat, sugars, icings, honey, toppings, artificial sweeteners, bottled and canned sauces, gravy, yeast extracts, spreads, dips, herbs and spices, jam and marmalade, pulses
