# Supplementary material for: Food price elasticity estimates in Australia
Source: Nat Food. 2025 Jul 11;6(7):725–32. doi: 10.1038/s43016-025-01184-1 (PMC12283402; doi:10.1038/s43016-025-01184-1)
Supplement: Supplementary file 2 — Reporting Summary [file 43016_2025_1184_MOESM2_ESM.pdf]

nature portfolio

Corresponding author(s): Tazman Davies

Last updated by author(s): May 11, 2025

Reporting Summary

Nature Portfolio wishes to improve the reproducibility of the work that we publish. This form provides structure for consistency and transparency in reporting. For further information on Nature Portfolio policies, see our Editorial Policies and the Editorial Policy Checklist.

Statistics

For all statistical analyses, confirm that the following items are present in the figure legend, table legend, main text, or Methods section.

n/a	Confirmed	
☐	☒	The exact sample size (*n*) for each experimental group/condition, given as a discrete number and unit of measurement
☐	☒	A statement on whether measurements were taken from distinct samples or whether the same sample was measured repeatedly
☐	☒	The statistical test(s) used AND whether they are one- or two-sided<br>*Only common tests should be described solely by name; describe more complex techniques in the Methods section.*
☐	☒	A description of all covariates tested
☐	☒	A description of any assumptions or corrections, such as tests of normality and adjustment for multiple comparisons
☐	☒	A full description of the statistical parameters including central tendency (e.g. means) or other basic estimates (e.g. regression coefficient) AND variation (e.g. standard deviation) or associated estimates of uncertainty (e.g. confidence intervals)
☐	☒	For null hypothesis testing, the test statistic (e.g. *F*, *t*, *r*) with confidence intervals, effect sizes, degrees of freedom and *P* value noted<br>*Give P values as exact values whenever suitable.*
☒	☐	For Bayesian analysis, information on the choice of priors and Markov chain Monte Carlo settings
☒	☐	For hierarchical and complex designs, identification of the appropriate level for tests and full reporting of outcomes
☒	☐	Estimates of effect sizes (e.g. Cohen's *d*, Pearson's *r*), indicating how they were calculated

Our web collection on statistics for biologists contains articles on many of the points above.

Software and code

Policy information about availability of computer code

Data collection	This study relied on five years of food purchasing data in the Australian NielsenIQ Homescan dataset (i.e., 2015 - 2019). The data is proprietary.
Data analysis	Analyses were conducted using R v 4.4.1 and the micEconAids library.

For manuscripts utilizing custom algorithms or software that are central to the research but not yet described in published literature, software must be made available to editors and reviewers. We strongly encourage code deposition in a community repository (e.g. GitHub). See the Nature Portfolio guidelines for submitting code & software for further information.

Data

Policy information about availability of data

All manuscripts must include a data availability statement. This statement should provide the following information, where applicable:

- Accession codes, unique identifiers, or web links for publicly available datasets
- A description of any restrictions on data availability
- For clinical datasets or third party data, please ensure that the statement adheres to our policy

The authors do not have permission to share data.

Research involving human participants, their data, or biological material

Policy information about studies with human participants or human data. See also policy information about sex, gender (identity/presentation), and sexual orientation and race, ethnicity and racism.

Reporting on sex and gender	We report price elasticities at the population level and for different socio-economic quintiles. Unfortunately, we were not able to stratify analyses by sex or gender as the NielsenIQ homescan dataset contains food purchases at the household level and the not individual level.
Reporting on race, ethnicity, or other socially relevant groupings	We report price elasticities for different socio-economic quintiles. Households were categorised using the Index of Relative Social Advantage and Disadvantage (IRSAD) as defined by the Australian Bureau of Statistics. This index summarises the economic and social conditions of households living within a particular area by considering a range of indicators such as income levels, education levels, employment rates, and housing. In our analyses, we controlled for confounding by consideration of the following variables: region, time, and household size.
Population characteristics	See section 'Behavioural & social sciences study design"
Recruitment	This study did not involve recruiting participants. NielsenIQ recruits participants using an online application process.
Ethics oversight	This study was approved by the University of New south Wales Human Research Ethics Committee (HC200244)

Note that full information on the approval of the study protocol must also be provided in the manuscript.

Field-specific reporting

Please select the one below that is the best fit for your research. If you are not sure, read the appropriate sections before making your selection.

☐ Life sciences ☒ Behavioural & social sciences ☐ Ecological, evolutionary & environmental sciences

For a reference copy of the document with all sections, see nature.com/documents/nr-reporting-summary-flat.pdf

Behavioural & social sciences study design

All studies must disclose on these points even when the disclosure is negative.

Study description	The study is quantitative, both cross-sectional and longitudinal
Research sample	The research sample is a panel of households within the Australian NielsenIQ Homescan dataset. The number of included households in the NielsenIQ Homescan dataset was 6969 in 2015, 7348 in 2016, 7427 in 2017, 7373 in 2018, and 7535 in 2019, totalling 36,697 household-years across the study period. This involved 10,008 unique households, with approximately half (n = 4854) appearing in all five years of data, and 1263, 1168, 1148, and 1575 appearing in four years, three years, two years, and one year of data, respectively. Households were based in Melbourne (20%), Sydney (19%), Brisbane (14%), Perth (8%), Adelaide (7%), and other areas (31%). The mean household size was 2.7 persons. Each household recorded all packaged and unpackaged foods purchased for at-home consumption (i.e., from supermarkets, convenience stores, and grocers).
Sampling strategy	NielsenIQ recruited households using an online application process. To ensure the panel was representative of Australia's geographic distribution, NielsenIQ used quotas for different geographical segments to avoid clustering of households by location. They also controlled for other factors that are relevant to grocery shopping, such as household size, lifestage, and income level.
Data collection	To collect data, households used a portable barcode scanner to scan product barcodes (for packaged items) and a scanning guide booklet (for unpackaged items such as fresh fruits and vegetables) for all foods and beverages purchased for at-home consumption. For each scanned product, panel members manually recorded the price paid and the quantity purchased. NielsenIQ then determined each product's name, brand, package size (kg or L), and food category via linkage with a master product dataset. No data was collected on products purchased for consumption outside of the home (e.g., from restaurants and cafes)
Timing	Data were collected between January 2015 and December 2019
Data exclusions	Households that were flagged by NielsenIQ for containing unreliable purchase information were excluded from the study. For each calendar year, households were flagged if they (i) were not on the panel for the entire 52-week period, (ii) did not scan a barcode for at least 26 weeks, or (iii) did not meet the minimum spend threshold (i.e., an average of AU$5 per week).
Non-participation	Participants that dropped out of the study were excluded, as noted above.
Randomization	Participants were not randomized into groups.

Reporting for specific materials, systems and methods

We require information from authors about some types of materials, experimental systems and methods used in many studies. Here, indicate whether each material, system or method listed is relevant to your study. If you are not sure if a list item applies to your research, read the appropriate section before selecting a response.

Materials & experimental systems

n/a	Involved in the study
☒	☐ Antibodies
☒	☐ Eukaryotic cell lines
☒	☐ Palaeontology and archaeology
☒	☐ Animals and other organisms
☒	☐ Clinical data
☒	☐ Dual use research of concern
☒	☐ Plants

Methods

n/a	Involved in the study
☒	☐ ChIP-seq
☒	☐ Flow cytometry
☒	☐ MRI-based neuroimaging

Plants

Seed stocks	*Report on the source of all seed stocks or other plant material used. If applicable, state the seed stock centre and catalogue number. If plant specimens were collected from the field, describe the collection location, date and sampling procedures.*
Novel plant genotypes	*Describe the methods by which all novel plant genotypes were produced. This includes those generated by transgenic approaches, gene editing, chemical/radiation-based mutagenesis and hybridization. For transgenic lines, describe the transformation method, the number of independent lines analyzed and the generation upon which experiments were performed. For gene-edited lines, describe the editor used, the endogenous sequence targeted for editing, the targeting guide RNA sequence (if applicable) and how the editor was applied.*
Authentication	*Describe any authentication procedures for each seed stock used or novel genotype generated. Describe any experiments used to assess the effect of a mutation and, where applicable, how potential secondary effects (e.g. second site T-DNA insertions, mosiacism, off-target gene editing) were examined.*
